# Dysmotility in Eosinophilic Esophagitis

**DOI:** 10.3389/fped.2022.853754

**Published:** 2022-02-28

**Authors:** Charmaine Chai, Usha Krishnan

**Affiliations:** ^1^Department of Pediatric Gastroenterology, The Children's Hospital at Westmead, Sydney, NSW, Australia; ^2^Department of Pediatric Gastroenterology, Sydney Children's Hospital, Randwick, Sydney, NSW, Australia; ^3^School of Women's and Children's Health, University of New South Wales, Sydney, NSW, Australia

**Keywords:** eosinophilic esophagitis, manometry, esophageal dysmotility, motility in children, dysphagia

## Abstract

Eosinophilic esophagitis (EoE) is an immune mediated chronic inflammatory disease resulting from antigen exposure and is characterized by mucosal inflammation with eosinophils. Diagnosis is based on the histological finding of at least 15 eosinophils per high power field in esophageal biopsy specimens from upper gastrointestinal endoscopies. These endoscopies are usually performed in the setting of esophageal dysfunction, however, EoE can occasionally be incidentally diagnosed during endoscopies performed for other indications like coeliac disease. The eosinophilia is in the absence of other causes of esophageal eosinophilia (e.g., parasitic infection, esophageal leiomyomatosis or Crohn's disease). Presentation can be wide ranging and often varies according to age. Infants and younger children can present with choking/gagging, feed refusal, failure to thrive, irritability and vomiting. Older children and adults commonly present with dysphagia, chest pain or food bolus obstruction. EoE was first described in the 1970s, but was only recognized as a distinct disease entity in the 1990s. It has been rising in incidence and prevalence, with reported prevalence ranging between 1 in 2,500 and 1 in 10,000. Although the diagnosis of EoE is dependent on clear histopathologic diagnostic criteria, there is a disconnect between the degree of esophageal eosinophilia and symptom severity especially that of reported dysphagia. Multiple anatomical changes can be seen in the spectrum of presentations of EoE which explain dysphagia, including isolated strictures, diffuse trachealisation, fixed rings, including Schatzki, as well as tissue remodeling and fibrotic changes. However, a majority of EoE patients do not have any of these findings and will still often report ongoing dysphagia. Some will report ongoing dysphagia despite histological remission. This suggests an underlying esophageal dysmotilty which cannot be assessed with endoscopy or correlated with histological changes seen in biopsies. This review will describe the types of motor disturbances seen and their prevalence, the pathophysiological basis of dysmotility seen in EoE, how best to investigate esophageal dysfunction in EoE and the role of manometry in the management of EoE.

## Types of Motility Patterns Seen in Eoe

**Figure 1:**
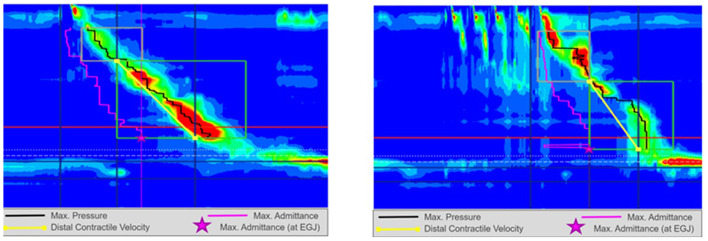
High resolution manometry in eosinophilic esophagitis showing normal motility on 5 ml wet swallow and multiple rapid swallow (5 × 2 ml) in an adult. Reprinted with permission from Prof. Taher Omari.

**Figure 2:**
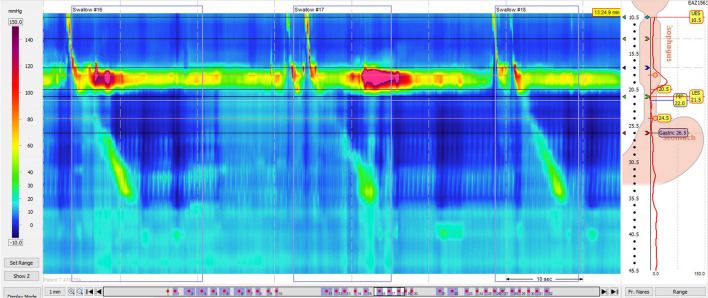
High resolution manometry in eosinophilic esophagitis showing weak peristalsis on wet swallows in a child. Reprinted with permission from Dr Rachel Rosen.

**Figure 3:**
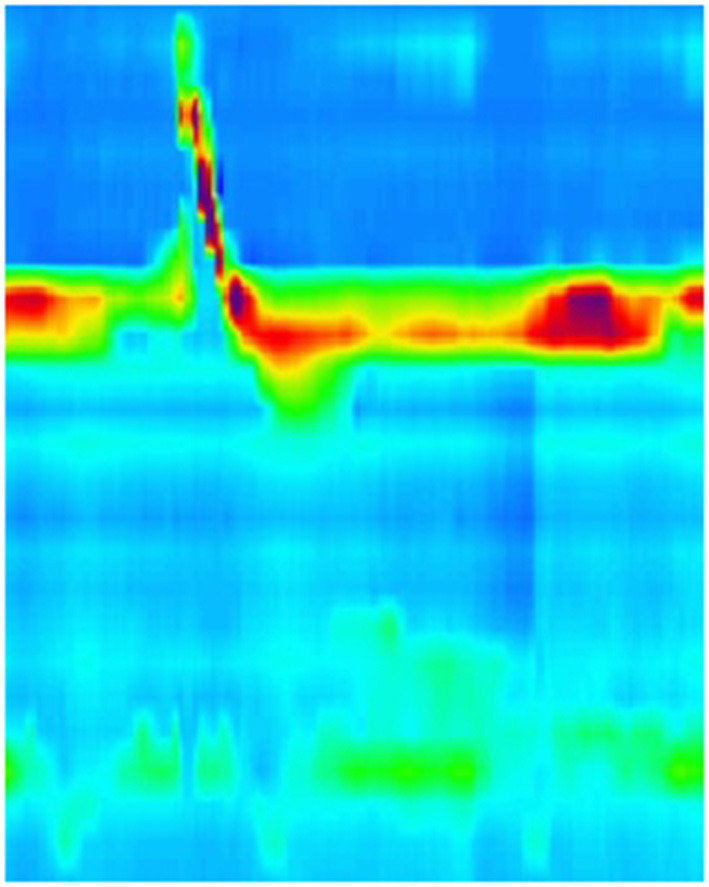
High resolution manometry in eosinophilic esophagitis showing weak peristalsis with wet swallow in an adult. Reprinted with permission from Prof. Taher Omari.

**Figure 4:**
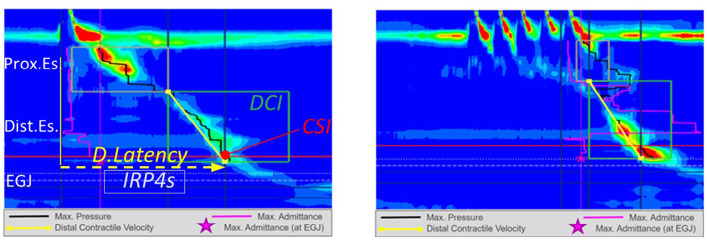
High resolution manometry in eosinophilic esophagitis showing ineffective esophageal motility on 5 ml wet swallow and multiple rapid swallow (5 × 2 ml) in an adult. Reprinted with permission from Prof. Taher Omari.

**Figure 5:**
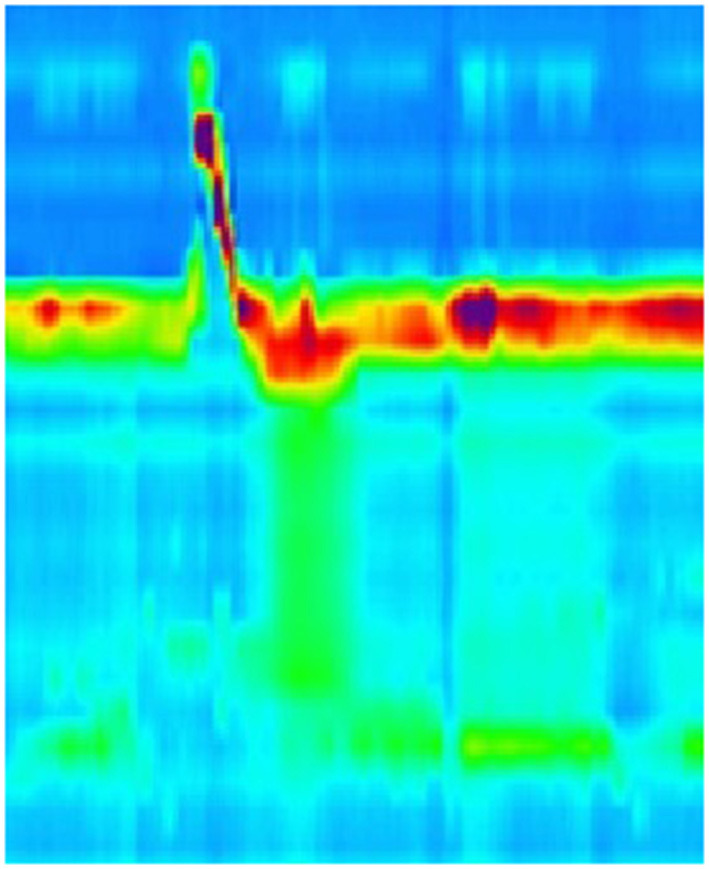
High resolution manometry in eosinophilic esophagitis showing pan-esophageal pressurization with wet swallow in a child.

The pathogenesis of dysphagia in EoE remains elusive, with multiple theories about the cause of the dysphagia. Dysmotility has been thought to be causative, however, assessment of this has proven difficult mostly due to the fact that no single motility pattern has been associated with EoE. The results of both conventional and high resolution manometry studies in EoE groups have been diverse, ranging from normal peristalsis to hypo contractile patterns (Figures 1–3), including ineffective esophageal motility (IEM) (Figure 4) and absent contractility, as well as hyper contractile patterns such as distal esophageal spasm (DES), nutcracker esophagus, jackhammer esophagus and pan-esophageal pressurization (Figure 5). Esophago-gastric junction outflow obstruction and achalasia have also been described. It has been hypothesized that the different phases in the development of esophageal motor abnormalities in EoE may reflect a progression of disease from normal to hyper peristalsis/spastic to low amplitude simultaneous contractions, followed by ineffective esophageal motility and eventually leading to aperistalsis in severe cases.

Attwood et al. was first to describe a cohort in 1993 of patients with what is now known as EoE and identified the variability of manometric findings (1). Attwood et al. described a group of 12 individuals presenting with episodic dysphagia who all had esophageal hyper eosinophilia (defined as >20 per high power field) and normal esophageal acid exposure on 24 h pH monitoring. Within this group two had nutcracker esophagus, two had diffuse spasm while seven others had hypo motile changes. There have been multiple review articles which have confirmed the diversity of motility presentations in EoE. A review article published in 2008 by Nurko and Rosen identified 22 published series or case reports of esophageal manometry in EoE patients (2). This reports identified 144 patients, 29 of whom were children. Primary motility disorders were found in only 12 adult patients with EoE, 2 of whom had achalasia, 7 with diffuse esophageal spasm and 3 with nutcracker esophagus. Non-specific peristaltic abnormalities (tertiary contractions, low amplitude and ineffective peristalsis) were reported in 42 patients (35 adults and 7 children) and 11 adults had high amplitude contractions. Overall, 59 (41%) of patients had an abnormal esophageal manometry. A systematic review published by Furuta et al. in 2007 identified 10 studies which had reviewed esophageal manometry in EoE patients. 41/77 (53%) of adults had abnormalities, however, all 14 children included had normal manometry (3). A more recent review by Weiss at al was published in 2015, which included 15 studies with a total of 387 patients (4). The occurrence of abnormal esophageal manometry varied widely from 4 to 87% of patients. Weiss et al. suggested that these inconsistencies could be explained by variable disease activity at the time of the study or disease duration, but also noted that it is unclear whether there was a correlation between dysmotility and symptoms.

## Pathophysiological Basis of Dysmotility in Eoe

The pathogenesis of EoE is complex, and likely can be attributable to a number of inflammatory pathways. Cellular mediators have an important role in the development of dysmotility in EoE. Exposure to food or aero allergens induces a Th2 response, attracting eosinophils to the esophagus. Eosinophils secrete products that can either excite or relax esophageal muscle, which may explain the variety of motility abnormalities observed. Leukotriene D3, prostaglandin F2α and thromboxane B2 all cause esophageal muscle to contract, while IL-6 and IL-13 cause relaxation (5–7). Cao et al. detected pro-inflammatory cytokines like IL-6 and IL-1beta in the circular smooth muscles of the esophagus which reduce esophageal contraction by inhibiting acetylcholine esterase release in the myenteric neurons (8). Cytoskeletal protein synaptopodin (SYNPO) may also have a role as it is expressed in esophageal epithelium and up-regulated by IL-13 in EoE. SYNPO is co-localized with actin filaments and regulates esophageal epithelial cell motility and barrier integrity (9).

Eosinophils degranulation also contributes to dysmotility, as eosinophils contain toxic granular proteins, including major basic protein (MBP), eosinophil cationic protein (ECP) eosinophil-derived neurotoxin (EDN) and eosinophil peroxides (EPO) (10). These proteins are all pro-inflammatory and can affect esophageal motility. EDN and ECP both have ribonuclease activity which can result in axonal necrosis. While MBP also activates muscarinic M2 acetylcholine receptors which are responsible for smooth muscle contraction in the distal two thirds of the esophagus (10–15). In addition to this, MBP also triggers degranulation of basophils and mast cells, which also are significant in the pathogenesis of EoE.

Mast cells are increasingly recognized as significant in the pathogenesis of EoE. Mast cell genes are up regulated in EoE and lead to an increase in pro inflammatory mediators like TNFα/β and tryptase which can result in type IV collagen production and fibrosis impacting on motility (16, 17). Activation of Acetylcholine (Ach) by histamine released from mast cells in esophageal wall may cause contractions of smooth muscle fibers in the muscularic mucosa resulting in uncoordinated contractions (18). A study by Aceves et al. (19), showed that mast cells rather than eosinophils infiltrate esophageal smooth muscle in patients with EoE. These cells express TGFβ1 which increased human esophageal smooth cell contractility in *in vitro* studies. Mast cell degranulation releases tryptase and histamine which can activate smooth muscle contraction pathways, as well as other pro-inflammatory and cytotoxic mediators which can cause loss of enteric neurons.

Both eosinophils and mast cells express transforming growth factor β1 (TGFβ1) which increased human esophageal smooth muscle contractility in *in vitro* studies and induced tissue fibrosis (20). Activated eosinophils also have a role in fibroblast proliferation and collagen deposition, with secondary fibrosis through secretion of Th2 cytokines, TGFβ1 and other eosinophil products. These may result in esophageal wall rigidity and contractile dysfunction (7, 17). Induction of tissue fibrosis may be compounded by the secretion of IL-13, IL-8 and vascular endothelial growth factor (VEGF) by eosinophils, may lead to tissue remodeling and alter motility as it does in scleroderma (21). Epithelial mesenchymal transformation (EMT) has also been implicated in the pathogenesis of fibrosis seen in EoE, which can impact esophageal wall compliance and motility (22).

Spechler postulated in 2019 that a possible mechanism for dysmotility in EoE is the release of cytotoxic secretory products destroying intramural neurons, and that achalasia may be a muscle-predominant form of EoE (21). The terminology “eosinophilic esophagitis” first appeared in the literature in 1978 in relation to a patient with achalasia who had eosinophil infiltration in biopsies of the esophageal muscularis propria (23). There are several similar cases reports, where patients have presented with significant esophageal obstruction or wall thickening with mild mucosal eosinophilia but dense infiltration of eosinophils in the deep muscle layers. This theory is supported by a case series of 28 patients with achalasia by Jin et al. in 2018. Eighty six percentage of these patients had eosinophilic infiltrates with positive staining for MBP and EDN identified in muscularis propria specimens taken at the time of Per Oral Endoscopic Myotomy (POEM) for hypercontractile motility disorders (24). Notably, no mucosal eosinophils were identified.

Given this, it is possible that there are different presentations of eosinophilic esophagitis depending on depth of gastrointestinal tract infiltration, much like eosinophilic gastroenteritis. This may explain why some patients complain of ongoing dysphagia despite resolution of mucosal eosinophilia and may have therapeutic implications as some current treatments such as topical steroids and proton pump inhibitors (PPIs) only aim at correcting mucosal eosinophilia and not eosinophilia in the esophageal muscles. Assessment of this theory is difficult given the complexity of obtaining deep esophageal biopsies, and it is unclear whether the eosinophils seen infiltrating the esophageal muscularis propria in these cases are the cause of enteric neuronal destruction or merely a response to their destruction.

## Assessment of esophageal Dysfunction in Eoe

### Barium Contrast Study

There is currently no role for barium esophagogram for evaluation of esophageal motility or distensibility in EoE patients. Lee et al. observed heterogeneity in their cohort of EoE patients, and that only ~50% of patients had abnormal baseline esophageal diameters. They also found that significant changes in diameter had no correlation to clinical outcomes, indicating no role in ongoing management either (25).

### Esophageal Manometry

Esophageal manometry, particularly high resolution manometry, has been useful in understanding dysmotility in EoE patients, but disappointingly no distinct manometric findings have been identified. With the advent of the Chicago 4 classification of manometry patterns (Ref), the patients previously classified as having weak and frequent failed peristalsis would now be classified as having Ineffective Esophageal Motility (IEM) and those having Hypertensive Peristalsis as having Distal Esophageal Spasm (DES) (26). Multiple studies have published manometric data from EoE patients, using both conventional and high resolution manometry (Tables 1, 2). Notably, apart from the studies by Cheung and Nurko et al, all of the studies were on adult EoE patients. In studies by both Van Rhijn et al. and Roman et al, no significant differences in pressure topography parameters between EoE vs. gastroesophageal reflux disease (GERD) patients were observed. Roman's group observed that EoE patients were more likely to have abnormal bolus pressurization patterns (compartmentalized in 19% and pan-esophageal in 17%) during swallows (27, 28). Early pan-esophageal pressurization in conjunction with normal esophago-gastric junction (EGJ) relaxation was a finding specific to EoE. The authors found that increasing the volume of the bolus challenge increased the number of swallows with pressurization in EoE patients. This is possibly due to exaggerated longitudinal muscle (LM) contraction, causing reduced esophageal compliance, or may be secondary to obstructive findings on endoscopy.

**Table 1:** Conventional manometry studies in eosinophilic esophagitis.

**References**	**Study type**	**No. of patients with EoE/No. of controls**	**Peristaltic changes (%)**	**Normal (%)**
Attwood (1)	Retrospective	12/90 (GERD)	DES 2 (17%) “Nutcracker” 2 (17%) Reduced peristalsis 7 (58%)	2 (17%)
Vitellas (38)	Retrospective	13/0	DES 1 (8%) Prolonged peristalsis 1 (8%)	10 (77%)
Cheung (39)	Retrospective	11 children/6 (dysphagia)	None	11 (100%)
Croese (40)	Retrospective	13/0	Nonspecific changes 5 (38%)	8 (62%)
Remedios (41)	Prospective	23/0	Aperistalsis 1 (4%)	22 (96%)
Gonsalves (42)	Retrospective	15/0	DES 1 (7%) Nonspecific disorders (60%)	5 (33%)
Lucendo (43)	Prospective	29/0	Hypoperistalsis 17 (58%) High amplitude contractions 9 (31%)	3 (10%)
Lucendo (44)	Retrospective	12/0	Nonspecific changes 6 (50%) Distal hyperkinetic peristalsis 3 (25%) Simultaneous contractions 1 (8%)	2 (17%)
Korsapati (45)	Prospective	10/10	None	10 (100%)
Nurko (37)	Prospective	17 (children)/24 (13 GERD, 11 healthy)	Peristaltic changes 7 (41%)	10 (59%)
Bassett (34)	Prospective	30/0	Nonspecific changes 5 (16%) Amplitude > 180 mmHg 2 (7%)	23 (77%)
Hejazi (2010)	Retrospective	14/0	“Nutcracker” 2 (14%) Nonspecific disorder 2 (14%) Aperistalsis 2 (14%)	6 (43%)
Moawad (36)	Retrospective	75/0	Ineffective peristalsis 25 (33%) “Nutcracker” 3 (4%)	47 (63%)
Monnerat (33)	Retrospective	20/0	Ineffective peristalsis 3 (15%)	15 (75%)

*GERD, Gastro-Esophageal Reflux Disease*.

**Table 2:** High resolution manometry studies in eosinophilic esophagitis.

**References**	**Study type**	**No. of patient with EoE/No. of controls**	**Peristaltic changes (%)**	**Normal (%)**
Martin (35)	Prospective	21/21 (GERD with dysphagia)	Reduced peristalsis 6 (28%) Pan-esophageal pressurisation 10 (48%)	5 (25%)
Roman (27)	Retrospective	48/98 (48 GERD, 50 healthy)	EGJOO 1 (2%) Aperistalsis 1 (2%) Hypercontractility 1 (2%) Rapid contractions 2 (4%) Common interrupted peristalsis 5 (10%) Reduced peristalsis 8 (17%)	30 (63%)
Van Rhijn (28)	Prospective	31/62 (31 GERD, 31 healthy)	Reduced peristalsis 27% Interrupted peristalsis 12%	13 (42%)
Nennstiel (30)	Prospective	20/0	Early pan-esophageal pressurisation 3 (15%) Compartmentalised esophageal pressurisations 1 (5%) Frequently failed peristalsis 1 (5%) Weak peristalsis 2 (10%)	13 (65%)
Colizzo (29)	Retrospective	29/0	Jackhammer esophagus 2 Weak peristalsis 2 EGJOO 1 Hypertensive LES 1	23 (80%)
Von Arnim (31)	Prospective	24/23	Hypomotility 8 EGJOO 5	11 (43%)

*GERD, Gastro-Esophageal Reflux Disease; DES, Diffuse Esophageal Spasm; EGJOO, Esophageal Gastric Junction Outlet Obstruction*.

It has been postulated that there are distinct phenotypes, with a study in 2016 by Colizzo et al. dividing EoE patients into a fibrostenotic (FS) subtype or inflammatory (IF) subtype depending on endoscopic findings and the patients undergoing manometry to assess for differences, with a focus on intrabolus pressure. Elevated IBP indicates abnormal resistive forces and is an objective measure of the pressure a bolus encounters as it moves down the esophagus and has been shown to correlate with dysphagia in other esophageal disorders. Colizzo found that intrabolus pressure (IBP) was higher in the fibrostenotic group (29). An IBP of 16 mmHg had a sensitivity and specificity for FS disease, of 70.5 and 75% respectively, to distinguish between the groups. This finding has not been replicated in subsequent studies (30). In a study done by von Arnim et al. (31), on 26 EoE patients vs. 23 controls, none of the HRM parameters including IBP showed no differences according to EoE subtype (FS or IF). This may be due to a difference in cohort, as all patients had strictures in this study, unlike the Colizzo study where all patients were non obstructive on endoscopy. There was also no statistically significant difference in IBP between FS and IF subtypes of EoE in the high resolution manometry in patients with EoE (HIMEOS) study, though the IBP was higher in the FS subtype (30).

Correlation between pH metry and manometry findings in EoE has also been an area of interest. Higher esophageal acid exposure time and lower baseline impedance values were significantly associated with eosinophilic infiltration in a study on 63 EA patients with EoE in a study by Pesce et al. (32). Monnerat and her group published the first study to correlate pH metry with manometry in 20 patients (33). Abnormal acid reflux index was seen in 25%, however no correlation was seen between abnormal reflux and presence of manometry changes.

There are numerous theories as to why multiple studies utilizing conventional and high resolution esophageal manometry, including the HIMEOS study, have not found a correlation between symptoms and motility patterns (2, 3, 28, 30, 31, 34–36). Studies have involved small numbers of patients with varying disease duration and have used varying definitions of motility disorders as well as varying methodologies to compare symptoms to manometric findings, making reviewing and comparing the studies difficult. In addition to this, there is no validated dysphagia scoring system for EoE. The natural history of EoE is also poorly understood and there is likely an evolution of the cause of dysmotility underlying EoE over time, with an evolving manometric pattern. Motor disorders may also represent an epiphenomenon. Symptom reporting may also be impacted by visceral hyperalgesia, abnormalities in central processing, esophageal hypervigilence and psychosocial factors.

Nurko et al. hypothesized that the lack of correlation may be due to the intermittent nature of dysphagia and that stationary manometry is unlikely to capture the relevant assessment of esophageal physiology at time of dysphagia. To address this, a study was conducted involving 17 children with EoE, 13 with gastro-esophageal reflux disease (GERD) and 11 healthy controls (37). These pediatric patients underwent both stationary high resolution manometry and a prolonged ambulatory esophageal manometry plus pH metry (PEMP) for 24 h. Forty one percentage of EoE patients had peristaltic changes during their stationary high resolution manometry, which is similar to previously published data. However, during PEMP, 76% of EoE patients had recorded abnormal motor function with every episode of dysphagia, indicating that in the pediatric population, dysphagia does correlate with manometric changes. Manometry changes in this study consisted of ineffective peristaltic waves, higher amplitude peristalsis and isolated contractions. This study did not evaluate whether EoE treatment resulted in an improvement of esophageal motor function. In the absence of impedance measurement there was also no objective evidence that motility abnormalities resulted in abnormal bolus transit.

Possible correlation between manometry findings and eosinophil count on histology has also been assessed. Bassett et al. (34), used conventional manometry and evaluated 32 adult patients, dividing them into normal and abnormal manometry findings. They found that eosinophil count on biopsy were similar in both groups. Similar findings were seen in study by Moawad et al. (36) where there was no significant difference in the mean peak eosinophil count amongst the different motility groups (normal/mild IEM/moderate IEM/severe IEM/nutcracker).

Manometric studies on EoE patients have shed some light on the natural history and development of dysmotility in EoE. Martin et al. found an association between pan-esophageal pressurization and a disease duration of >10 years, as well as a history of requiring endoscopic disimpaction (35). This was replicated in a study by Van Rhijn et al, which found the prevalence of motility disorders increased from 36% in those who had a disease duration of 5 years or less to 83% in those who had a disease duration of >16 years (28). The HIMEOS study did not show any statistical significance in disease duration in their cohort, but this may be due to the younger ages of their patients (30).

### Endoscopic Ultrasound

Whilst manometry is able to assess the circular muscle (CM), endoscopic ultrasound is of greater utility when assessing LM. Korsapati et al. in 2009 showed that LM may play an important role in dysphagia in EoE patients (45). In this prospective study, 10 EoE patients and 10 healthy controls underwent simultaneous high resolution manometry and EUS. Measurements were obtained before and after the use of edrophonium, which is an acetylcholinesterase inhibitor used to increase contraction amplitude. Muscle thickness was used as a surrogate marker for LM contraction, and this was found to be markedly diminished in EoE patients compared to controls. Asynchronicity between muscles layers was identified during peristalsis. Edrophonium had an effect on both controls and EoE patients, but this was more marked in controls. Notably, 3 patients who were on EoE treatment at the time showed a lower degree of LM dysfunction than other EoE patients.

This LM dysfunction may contribute to dysphagia through abnormal motility as well as the loss of coordination with CM, and may be due to inflammation LM from the release of cytokines and interleukins (IL-4, IL-5, IL-13 and eotaxin) by eosinophils and mast cells. The dysfunction in the LM may also be due to it not responding appropriately to cholinergic stimulation. Chronic inflammation of the LM could also induce scarring and fibrosis, thereby affecting movement of the esophagus in the longitudinal axis.

### EndoFLIP (Functional Luminal Imaging Probe)

EndoFLIP (Functional Luminal Imaging Probe) is a novel investigation which utilizes high resolution planimetry to generate three-dimensional images of the intraluminal esophageal anatomy during volumetric distension. This allows for objective measurement of tissue remodeling and fibrosis. EoE patients exhibit reduced esophageal distensibility and compliance secondary to esophageal wall thickening, edema and fibrosis. This may explain the presence of persistent symptoms despite inflammatory resolution due to structural remodeling of the esophagus which does not uniformly respond to EoE treatment.

EndoFLIP has been used in several studies with EoE patients. A study in 2011 by Kwiatek et al. found significantly reduced distensibility in EoE patients compared to controls, though this was independent to degree of esophageal eosinophilia (46). A study by Nicodeme investigated whether EndoFLIP was useful in EoE patients to identify susceptibility to food impaction/need for dilation and they were able to conclude that distensibility <225 cm2 is a predictor of need for dilation (47). No correlation was seen between esophageal distensibility and degree of eosinophilia. This lack of correlation could be because the reduced distensibility is secondary to fibrosis rather than mucosal hyper eosinophilia or secondary to deeper muscle wall involvement. The authors felt that esophageal eosinophilia is not predictive of outcome and correlates poorly with esophageal distensibility.

More recently, Carlson et al. postulated that there may be an association between EoE disease activity and esophageal contractile response (CR) to distensibility, also known as secondary peristalsis (48). In this retrospective study, the FLIP of 199 EoE patients were reviewed and assigned CR patterns (49). This was then compared against the endoscopic furrows and total endoscopic reference scores (EREFS) (50). This study found that while 34% of EoE patients had normal CR, there was a correlation between abnormal CRs and reduced esophageal distensibility, greater total EREFS and a greater duration of symptoms. Mucosal eosinophilia was also assessed and was similar between those with normal CR and abnormal CR. The authors concluded that fibrostenotic remodeling and evidence of esophageal obstruction lead to dysmotility in EoE, rather than the degree of eosinophilic inflammation.

A pediatric study by Hassan et al. used EndoFLIP to compare 11 EE and 12 control patients. They found that EoE subjects had significantly lower esophageal compliance and this correlated with epithelial remodeling severity (51). There was a correlation between esophageal eosinophilia and both decreased compliance and distensibility, which is different to the adult data. EREFS also correlated significantly with decreased compliance and distensibility. The authors concluded that compliance was a more sensitive gauge of altered esophageal biomechanics as it took into account the entire esophagus rather than just the narrowest point, and could better identify patients with a rigid but not narrowed esophagus. Study limitations included the fact that EoE patients were on different medications and at different disease duration and hence study could not assess relationship between disease duration, therapy type, distensibility and compliance.

### Effect of EoE Treatment on Esophageal Dysmotility

Although studies assessing motility abnormalities have not always shown correlation between mucosal eosinophilia and dysmotility, resolution of motility abnormalities (both hyper and hypo contractility) along with improvement in dysphagia with EoE treatment has been shown in multiple studies which strongly suggest causality (Table 3). Notably, in 2011 Savarino et al. published a case report of a patient with dysphagia and the manometric finding of achalasia, who had >50 eosinophils per HPF, (55) the symptoms and manometric findings resolved with the use of prednisolone, supporting the hypothesis that some cases of achalasia may have an underlying diagnosis of EoE and be responsive to EoE therapies.

**Table 3:** Manometry findings before and after eosinophilic esophagitis treatment.

**References**	**N**	**Treatment**	**Manometric findings before treatment**	**Manometric findings after treatment**
Landres et al. (23)	1	Myotomy	Vigorous achalasia	Normalised peristalsis and LES pressure
Hempel (52)	1	Systemic steroids	Low LES and DES	Low LES; normalised peristalsis
Lucendo (43)	1	Fluticasone	Hypomotility	80% normalised
Lucendo (44)	12	Fluticasone	High amplitude contractions in 3, severe abnormal peristalsis and 1 with mildly abnormal peristalsis	7 had ongoing manometric abnormalities but all improved
Nennstiel (30)	20	Budesonide	Early pan-esophageal pressurisation 3 (15%) Compartmentalised esophageal pressurisations 1 (5%) Frequently failed peristalsis 1 (5%) Weak peristalsis 2 (10%) Elevated IBP in 20%	Reduction of IBP in 55% of patients Resolution in 6/7 patients with manometric findings (no improvement in frequently failed peristalsis)
Tanaka (53)	1	Systemic steroids Myotomy	Jackhammer esophagus	No change after steroids; resolution after myotomy
Funaki (54)	3	Systemic steroids	Jackhammer esophagus	All normalised

*LES, Lower esophageal sphincter pressure; DES, Distal esophageal spasm; IBP, Intrabolus pressure*.

A larger retrospective study by Ghisa et al. was conducted to evaluate the possible association between EoE and obstructive esophageal disorders. The HRM of 109 patients with new diagnosis EoE were reviewed; 41 patients were found to have a motor disorder, amongst whom eight had achalasia (56). Three of the eight patients responded to steroid therapy based on symptoms and histology, and did not require more invasive intervention for management of achalasia. Unfortunately, HRM after EoE therapy was only performed in 1 of these patients, who was found to have ongoing EGJOO.

In the HIMEOS-study, symptomatic EoE patients were evaluated with HRM before and after 8 weeks of topical treatment with budesonide slurry (30). Primary endpoint of this study was the effect of treatment on the IBP, which reduced in 55% post therapy but was not statistically significant. The authors felt this was due to IBP not being an optimal parameter for the monitoring of successful treatment response in EoE patients. Other study limitations included the fact that in this study there were only low numbers of FS type patients and only 5 ml swallows were used for testing unlike the Colizzo et al. study.

## EoE in Esophageal Atresia

Recently there have several publications reporting a significantly higher prevalence (up to 17%) of EoE in patients with repaired esophageal atresia (EA). Certain genes which have an effect on esophageal motility have been found to be dysregulated in EoE patients with and without EA. These genes include ANO1, expressed by interstitial cells of cajal (ICC) and which governs SM contractions, and SYNPO2 (cytoskeletal protein synaptopodin), which is co-localized with actin filaments and regulates esophageal epithelial cell motility and barrier integrity. EoE patients with EA were also found to have a more severe phenotype when compared to EoE patients without EA (57). Whether this was due to more significant dysmotility, decreased compliance and/or a greater proportion of the EoE in EA patients being of the FS subtype is currently not known. Significant reduction in dysphagia, food bolus impactions, reflux symptoms and strictures needing dilation was observed post treatment of EoE in EA patients in study by Chan et al. (58). However, whether this symptomatic improvement was due to improved inflammation and/or motility parameters is currently not known as there are currently no published studies evaluating motility in EA patients with EoE at baseline and post EoE treatment.

### Summary

1. Dysphagia and food impaction, which are common symptoms in EoE patients are more commonly due to abnormal esophageal motility and dispensability than from anatomical changes like strictures.
2. Dysmotility, as seen in EoE may progress from hyper contractility to hypo contractility disorders. These abnormalities most likely result from interactions between eosinophil's and mast cells within the esophageal microenvironment.
3. HRM has allowed definition of motility changes seen in EoE patients, but no specific manometry pattern for EoE has been identified. Prevalence of these changes increases with disease duration. Effect of EoE treatment on these manometric abnormalities has not been well evaluated. Even though some of the motility abnormalities may improve after treatment of EoE it is not clear whether this correlates with symptomatic improvement. Currently HRM is not considered to be essential for the diagnosis or to assess efficacy of treatment of EoE. Ambulatory manometry has demonstrated temporal association between dysphagia and abnormal motility patterns in a single pediatric study.
4. Based on current literature, HRM can explain dysphagia only in a few EoE patients. EndoFLIP and EUS might be complementary investigations with HRM to explain dysphagia in EoE patients. Targeting the allergic and inflammatory process should still be the primary focus for therapeutic interventions but treatment outcomes should also focus on improving compliance of the esophageal wall and resolving the mechanical obstruction that drives symptom severity.

### Future Perspectives

Although recognition and understanding of the dysmotility seen in EoE has continued to evolve, larger studies need to be undertaken to confirm whether manometric abnormalities result in abnormal bolus transit and to understand if severity of histologic findings correlates with manometric findings and/or dysphagia.

Possible future areas of research could include:

1. Prospective longitudinal large studies using HRM (Chicago 4) and pressure flow metrics in” Swallow Gateway” to determine if manometric abnormalities result in abnormal bolus transit and if severity of histologic disease correlates with manometric abnormalities and/or severity of dysphagia (26, 59).
2. HRM studies on EoE patients to look for signs of early and late disease and to monitor disease activity with pre and post treatment studies with validated EoE specific dysphagia scores (60) and endoscopic (EREFS) and histological scores (PEESS).
3. Delay in diagnosis has been identified as a risk factor for esophageal stricture formation. Prospective studies to determine if early diagnosis and prolonged treatment until there is resolution of motor abnormalities reduces stricture risk and improves patient outcomes.
4. There is mounting evidence that mast cells and TGFβ1 might function as a potential therapeutic targets that are involved in both esophageal remodeling and dysmotility resulting in dysphagia in EoE patients. Hence future studies could help determine whether targeted therapies resulting in reduction in mast cell numbers and TGFβ1 expression, are associated with improved peristalsis and reduced symptoms.

## Conclusion

Although the answer to the question “What is the clinical impact of manometry testing on EoE management?” is still not clear, measuring the biomechanics in EoE is important to help determine objective surrogate endpoints for therapeutic clinical trials, as reliance upon symptom scoring alone or eosinophil count for response to treatment may ignore the most important underlying mechanism for symptoms. Although targeting the allergic and inflammatory process should still be a primary focus for therapeutic interventions, treatment outcomes should also focus on improving the motility abnormalities, compliance of the esophageal wall and resolving the mechanical obstruction that drives symptom severity. The different phenotypes of EoE will likely require different approaches and assessment of esophageal biomechanics (motility and compliance), which could help tailor therapy.

## Author Contributions

CC prepared and drafted the manuscript. UK critically reviewed and edited the manuscript. All authors contributed to the article and approved the submitted version.

## Conflict of Interest

The authors declare that the research was conducted in the absence of any commercial or financial relationships that could be construed as a potential conflict of interest.

## Publisher's Note

All claims expressed in this article are solely those of the authors and do not necessarily represent those of their affiliated organizations, or those of the publisher, the editors and the reviewers. Any product that may be evaluated in this article, or claim that may be made by its manufacturer, is not guaranteed or endorsed by the publisher.
